# Understanding healthy eating and physical activity community‐centred behaviour change interventions for underserved populations: A mixed methods rapid review

**DOI:** 10.1111/bjhp.70043

**Published:** 2025-12-16

**Authors:** Jessica Marshall, Anne‐Marie Minihane, Stephanie T. Jong, Sarah Hanson, Shamima Akter, Nikki Garner, Wendy Hardeman

**Affiliations:** ^1^ University of East Anglia Norwich UK

**Keywords:** behaviour change, community, health inequalities, healthy eating, physical activity, underserved

## Abstract

**Purpose:**

Community behaviour change interventions are a promising strategy for addressing unhealthy eating and physical activity behaviours in underserved populations. This review explores these interventions' characteristics by focusing on behaviour change techniques, evaluates their behaviour change effectiveness and examines participant experiences.

**Methods:**

A mixed‐methods rapid review was conducted. Five databases and the grey literature were searched and supplemented by hand searching. Results were screened, assessed for methodological quality and data extracted using the Behaviour Change Techniques Ontology. A convergent segregated approach was used to synthesize the results.

**Results:**

Twenty‐one studies were included. Commonly used behaviour change techniques were social support, guidance on how to perform the behaviour and monitoring. Intervention effects on healthy eating and physical activity behaviour change were small, but outcomes that measured influences on behaviour change (e.g., social support) improved. Participants reported largely positive experiences, mostly attributed to the intervention's tailoring to the target population's contexts and the engagement and expertise of those who delivered the intervention.

**Conclusions:**

Evidence of direct behaviour change remains limited; there is stronger evidence for improvements in factors that influence behaviour change. Qualitative evidence highlights the value of tailoring interventions to participants' lives and using relatable, knowledgeable sources for delivery. This could enhance engagement and contribute to improved outcomes over time. Further research is needed on how contextual tailoring is implemented and how the characteristics of those delivering interventions influence effectiveness and experience. Findings support the potential of community‐centred approaches, but long‐term evaluations in underserved contexts are needed.

**Statement of Contribution**

**What is already known about this subject?**

- The prevalence of unhealthy eating and physical activity behaviours is higher in underserved populations (groups that have disproportionately higher health burdens).
- Community‐centred behaviour change interventions are a promising approach to addressing this prevalence, but little is known about their characteristics, effectiveness and participant experiences in underserved population contexts.
- We know interventions developed with a clear theoretical foundation in behaviour change, such as those employing specific behaviour change techniques (BCTs), may be more effective than those less grounded in such theories. But, this has not been explored in community‐centred behaviour change interventions that address healthy eating and/or physical activity behaviours in underserved populations.

**What does this study add?**

- This study shows there is stronger evidence for improvements in factors that influence behaviour change than direct behaviour change in this context.
- This study highlights the value of tailoring interventions to participants' lives and using relatable, knowledgeable sources for delivery.
- This study demonstrates that further research is needed in this field on how contextual tailoring is implemented and how the characteristics of those delivering interventions influence effectiveness and experience. This includes long‐term evaluations in underserved contexts.

## INTRODUCTION

Healthy eating (HE) and physical activity (PA) behaviours help prevent and manage non‐communicable diseases (NCDs), including dementia, heart disease, stroke, diabetes and several cancers (World Health Organization, 2024a, 2024b). The disparities in HE and PA behaviours are a major contributor to inequalities for underserved populations (Cleland et al., 2012; Laraia et al., 2017; Patel et al., 2017), who are disproportionately affected by NCDs and face significant structural and systemic barriers to health (Marmot, 2020). From 2010 to 2020, in the United Kingdom, the largest health disparities were seen in the most deprived 10% of neighbourhoods, with health inequalities between underserved populations and the general population increasing quite markedly, with life expectancy improving for the top 60%, but not for the bottom 40% (Marmot, 2020). Effective behaviour change interventions are required to address these disparities and improve HE and PA health outcomes for underserved populations, but understanding health inequalities is complex due to the diversity of underserved populations and the impact of context. Additionally, current evidence demonstrates that little is known or understood concerning the engagement, effectiveness or experiences of HE and PA behaviour change interventions for underserved populations (Koshoedo et al., 2015; Laraia et al., 2017; Linder et al., 2022; Patel et al., 2017).

What constitutes an underserved population is complex (NIHR, 2020). Furthermore, intersectionality, defined as a framework to address how people's experiences are shaped based on intersecting social identities (e.g., race, ethnicity and gender), is important to consider when using terms that single out individual identities (Crenshaw, 1991; Holman et al., 2021; Sokoloff & Dupont, 2005). This review uses the guidelines and examples listed by the NIHR‐INCLUDE project to define underserved populations as a context‐specific term that encompasses groups who experience lower inclusion in research and experience a high healthcare burden (e.g., cultural minorities, low income and long‐term health conditions) (NIHR, 2020).

International public health guidance and academic literature advocate the central role of community‐centred approaches and interventions to improve health outcomes and reduce health inequalities (NICE Guidelines (NG44), 2016; World Health Organization, 2020). This promising approach can be better understood through the analysis of community‐based interventions that target HE and PA behaviours, as they reflect how the familial, social, socio‐economic and cultural environments influence behaviour change (Weyers et al., 2010). This review uses the South et al. (2019) definition for community‐centred approaches, which prioritizes building community strengths, participation and empowerment to improve health and well‐being. Community‐centred approaches focus on the concepts of social connectedness, equity and collaboration between communities and services, ensuring that people have control, voice and active roles in shaping health actions and outcomes.

Based on a preliminary search of databases (PROSPERO, the Cochrane Library and Scopus), conducted in April 2024, no review, ongoing or published has synthesized the quantitative and qualitative evidence in this subject area. The search found four other reviews that aligned closest to this review's aims (Baskin et al., 2021; Cleland et al., 2012; Everson‐Hock et al., 2013; Gormley et al., 2022), highlighting the limited evidence, with one review attributing this to the small number of studies and the variability of outcome measures used (Everson‐Hock et al., 2013). Despite some of the reviews not having a specific focus on HE or PA interventions using a community‐centred approach, some weak evidence suggested that multi‐component adult group‐based interventions with theoretical frameworks were the most effective in increasing PA in socio‐economically disadvantaged communities (Cleland et al., 2012), particularly interventions that use social components (Baskin et al., 2021; Gormley et al., 2022). All the reviews highlight a gap in understanding the specific characteristics of these kinds of community‐centred interventions and how these characteristics relate to intervention effectiveness and participant experiences.

Therefore, this rapid review's objective is to identify characteristics of interventions that influence HE and PA behaviours, that use a community‐centred approach for underserved populations and report the effectiveness of the interventions and the experiences of participants. A rapid review is justified for this project due to time constraints and, unlike scoping or full systematic reviews, it allows for a focused examination of specific research questions (Garritty et al., 2021).

The primary review question is:

What are the behaviour change characteristics of community‐centred HE and PA approaches for underserved populations?

The sub‐questions are:

- What is the evidence that community‐centred HE and PA approaches are effective in improving eating and PA behaviour change outcomes for underserved populations?
- What are the experiences of underserved populations who engage in HE and PA behaviour change community‐centred interventions?

## MATERIALS AND METHODS

This review was registered with the International Register of Systematic Reviews (PROSPERO) (CRD42024572262) on 24 July 2024 and is reported using the Preferred Reporting Items for Systematic Review and Meta‐Analysis (PRISMA) 2020 statement checklist (Page et al., 2021) (see File S1). The methods of this review followed the Joanna Briggs Institute (JBI) guidance for conducting mixed methods reviews (Stern et al., 2020) and the Cochrane Rapid Review Methods Guidance (Garritty et al., 2021, 2024).

### Approach

This rapid review addresses questions of effectiveness (quantitative research) and experience (qualitative research) by combining quantitative and qualitative data into an integrated single synthesis (Shaw et al., 2014; Stern et al., 2020). To specify and classify intervention characteristics, this review uses the Behaviour Change Technique Ontology (BCTO) (Marques et al., 2023) to identify BCTs, which are defined as the smallest component of an intervention compatible with retaining the chosen active ingredients (Michie et al., 2016). The BCTO was selected to reflect the latest classification of BCTs in the behaviour change field. The BCTO encompasses significantly more BCTs than previous taxonomies (Michie et al., 2011, 2013), thus increasing comprehensiveness, and categorizes BCTs into hierarchical levels, separate entities and definitions (Marques et al., 2023). The highest level of the ontology (Level 1) contains the 20 higher‐level groups of BCTs, which share active content with the individual BCTs that are classified in the lower levels of the hierarchy (Marques et al., 2023). Extracting BCTs using the BCTO increases understanding of how HE and/or PA community‐centred interventions facilitate behaviour change in underserved populations, and what BCTs are commonly used in these interventions.

### Data sources

A comprehensive search was conducted across five databases: MEDLINE [Ovid], PsycINFO [EBSCO], CINAHL [EBSCO], Scopus [Elsevier] and Cochrane/CENTRAL [Cochrane Library]. Grey literature was searched via the CORE database and supplemented by a structured search in Google. All database searches were supplemented by hand searching the reference lists of included studies.

### Eligibility criteria

Study inclusion and exclusion criteria were based on the PICO (**P**opulation, **I**ntervention, **C**omparator, **O**utcome) (Richardson et al., 1995) and PICo (**P**opulation, Phenomenon of **I**nterest, **Co**ntext) (Stern et al., 2014) to develop criteria that accounted for outcomes associated with quantitative and qualitative study designs (Table 1).

**TABLE 1:** Inclusion and exclusion criteria using the PICO/PICo framework.

Criteria	Studies with quantitative components		Criteria	Studies with qualitative components	
	Inclusion	Exclusion		Inclusion	Exclusion
Study design	All empirical study designs (including quantitative, mixed methods, qualitative and feasibility studies). Mixed methods studies will only be considered if data from the quantitative or qualitative components can be clearly disaggregated. Grey literature (reports) January 2019 to May 2024 Studies conducted in G7 countries, the European Union, the European Economic Area, Australia and New Zealand. Studies in the English language only	Studies conducted outside G7 countries, the European Union, Australia and New Zealand. Reviews (rapid reviews, narrative reviews, scoping reviews, systematic reviews, meta‐analyses or ‘any other type of review’) Case studies Editorials Book chapters News Conference abstracts & proceedings	‘^a^’	‘ ’	‘ ’
**PICO**			**PICo**		
**P**opulation	Adults (18+ years) from underserved groups, either self‐identified or identified from the study, as a target group, living in the community (including supported accommodation)	Studies that do not specify terms related to underserved groups, based on the NIHR INCLUDE (2020) criteria. Adults residing in prisons, care/nursing homes Children (<18 years)	**P**opulation	‘ ’	‘ ’
**I**ntervention	**Behaviour change interventions** *that use a* **Community‐centred approach** (defined as) activities, groups or services in the voluntary, community and social enterprises sector which: mobilize assets within communities (skills, knowledge, time, resources) focus on promoting health and well‐being in community settings using non‐clinical strategies, as opposed to healthcare service settings and clinical methods advocate partnership working with individuals and groups who typically experience healthcare barriers assist in advocating control over individuals' health encourage participatory methods (active and influential role) for community members (South et al., 2019, p. 359) *which* have the stated aim, within the study, of influencing **eating behaviours** *and/or* **physical activity behaviours**	Any interventions that are not using a community‐centred approach, as defined by South et al. (2019). Any clinical interventions (e.g., use a healthcare service setting or clinical methods). Any interventions not set in the voluntary, community and social enterprises sector. Any interventions that do not have the stated aim, reported by the authors, of influencing or aiming to influence, eating or physical activity behaviours. Interventions that address eating disorders, for example, anorexia and bulimia	Phenomenon of **I**nterest	Studies investigating the experience, event or process of participating in eating or physical activity behaviour change interventions, that use a community‐centred approach (as defined by South et al., 2019) for underserved groups	(See quantitative intervention exclusion criteria)
**C**omparator	Quantitative studies with or without comparison to another intervention or pre–post intervention	(No restriction)	**Co**ntext	Qualitative studies that consider the cultural factors, geographical location, specific ethnic or gender‐based interests or setting of the participants or intervention	(No restriction)
**O**utcome	Any quantitative outcome measures related to intervention behaviour change effectiveness on the participants. Validated or unvalidated outcome measures (e.g., psychometric instruments and/or other standardized measures)	Outcome measures that are not related to behaviour change			

^a^ Speech marks indicate duplication of quantitative component criteria.

#### Study design

Studies could be any study design (quantitative, qualitative, feasibility, mixed methods) but excluded reviews, protocols, case studies, editorials, news, book chapters, conference abstracts or proceedings. Studies conducted in G7 countries, the European Union, the European Economic Area, Australia and New Zealand were included to reflect the United Kingdom's community healthcare context, which is the context of this review's community‐centred approach definition (South et al., 2019). Furthermore, these countries were chosen to have a manageable scope, whereby focusing on global regions where NCDs, rather than infectious diseases, are the primary public health concern meant reducing the heterogeneity of the identified research. Studies not published in the English language were excluded.

#### Population

Participants were adults (18+) who were identified as or self‐identified according to the characteristics of an underserved population, as defined by the examples in the NIHR‐INCLUDE project (NIHR, 2020). Studies that included adults and children (under 18 years of age) were included if data about the adult population could be extracted and analysed as a sub‐group. Studies reporting outcomes for underserved populations from informants were included if they explicitly referenced the inclusion of participants from underserved groups.

#### Intervention

This review only included interventions that used a community‐centred approach, as defined by South et al. (2019). Included studies contained the following components (South et al., 2019):

- mobilizing assets within communities (skills, knowledge, time and resources),
- focusing on promoting health and well‐being in community settings using non‐clinical strategies, as opposed to healthcare service settings and clinical methods,
- advocating partnership working with individuals and groups who typically experience healthcare barriers,
- assist in advocating control over individuals' health and
- encourage participatory methods (active and influential role) for community members.

Interventions were excluded when they were not set in the voluntary, community and social enterprise (VCSE) sector, defined as organizations that are independent of the government, that represent and advocate for the local community or groups with specific needs, and aim to promote social, economic, environmental or cultural benefits (Nield et al., 2023).

#### Outcomes

The main outcomes of interest were the characteristics of the interventions, evidence of improved HE and/or PA behaviours, and participant experiences. Studies with quantitative components that assessed outcomes on intervention effectiveness related to behaviour change influences (e.g., self‐efficacy, knowledge, attitude and capability) were included, recognizing that these influences directly impact behaviour change capability. Studies with qualitative components included participant experiences of the intervention and investigated contextual factors, including culture, geographical location, specific ethnic interests or the setting of the participants or intervention.

### Search strategy

**FIGURE 1:**
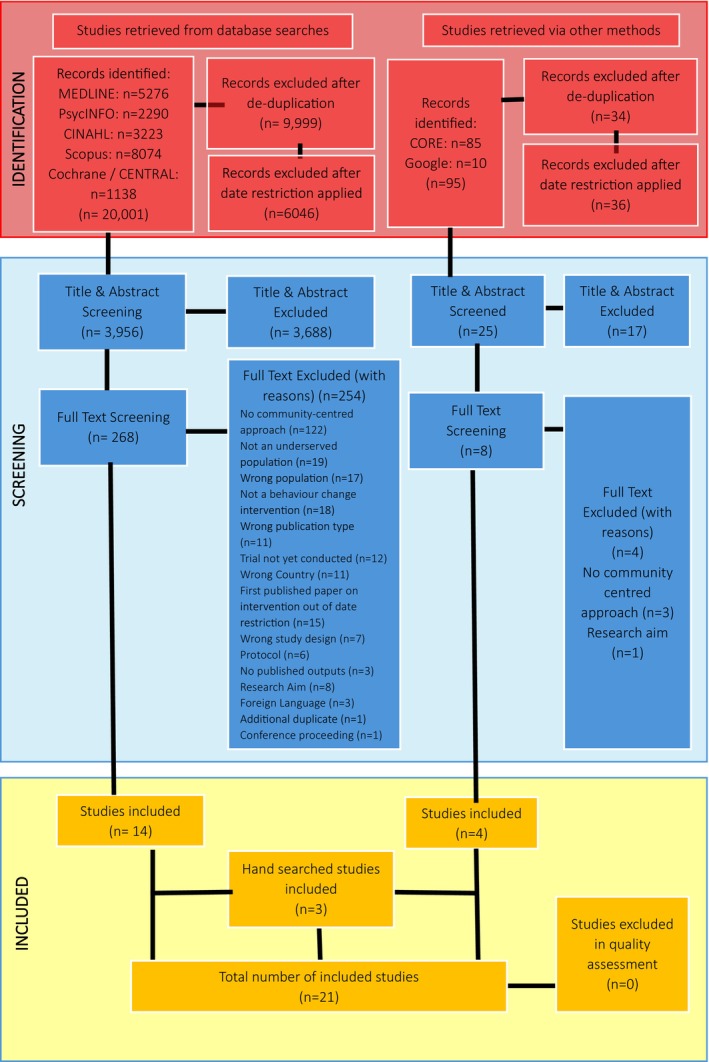
PRISMA flowchart.

The search strategy was developed in collaboration with an academic librarian and used relevant keywords and database‐specific terms for ‘eating’, ‘physical activity’, ‘community‐centred approach’ and ‘underserved’ (see File S2 for a copy of the search string). Originally, the search had no date limitation and ran from database inception to May 2024. During title and abstract screening, this was modified by the research team, where a 5‐year date restriction was applied, and only interventions that had their first empirical paper published after 1st January 2019 were included. The date restriction reflects the international pivot in recent years towards advocating community‐centred approaches to improve health outcomes and reduce health inequalities (NICE Guidelines (NG44), 2016; World Health Organization, 2020; Public Health England, 2015), and to reflect the period of availability of the South et al. (2019) definition of community‐centred approach. The application of the 5‐year date restriction is accounted for in the PRISMA diagram (Figure 1) and approved as an amendment to the study's PROSPERO registration.

### Study inclusion

Following de‐duplication in EndNote v.20, the records were imported into Rayyan for screening (Ouzzani et al., 2016). Title, abstract and full‐text screening of eligible studies were independently screened by the first reviewer, and a random sample of 10% was screened by the second reviewer at each stage, with high agreement of >80% consensus noted in each stage. Any disagreements were resolved by a third reviewer through consensus. Full consensus of the included studies was reached.

### Data extraction

Study characteristics related to BCTs were extracted using the BCTO data extraction template, where BCTs are categorized into levels (Norris et al., 2024), and BCT annotation guidelines. After the BCTO was piloted between two reviewers, BCTs were independently extracted across all intervention arms, with text highlighted where the BCTs were present by the first reviewer. In the first instance, BCTs were coded by Level 1 and then at Level 2 and beyond. A random sample of 10% was extracted, compared and checked by the second reviewer, with an inter‐rater reliability score calculated.

Behaviour‐related quantitative and qualitative outcomes were extracted using a spreadsheet template designed by the first author, which was piloted by two co‐authors, with adjustments made where necessary. Data were extracted independently by the first reviewer, and a random sample of 10% was checked by the second reviewer, with full agreement reached after consensus discussion.

### Quality assessment

Critical appraisal of each included study was carried out using the Joanna Briggs Institute Critical Appraisal Tools (JBI, 2024) and the Mixed Methods Appraisal Tool (MMAT) (Hong et al., 2018). Studies were independently appraised by the first reviewer, with a random sample of 10% checked by the second reviewer, with full agreement reached after consensus discussion. Additional information regarding evidence of ethical approval was sought and obtained from the corresponding authors of two included studies via email.

### Data synthesis

A narrative synthesis (Popay et al., 2006) was conducted that aligned the data to the review questions and reported results descriptively or thematically. A convergent segregated approach was undertaken to first separately synthesize the quantitative and qualitative data and then integrate them (Lizarondo et al., 2024; Stern et al., 2020). A convergent segregation approach involves independently synthesizing quantitative and qualitative data, resulting in the generation of separate quantitative and qualitative evidence that is later integrated (Stern et al., 2014). In this study, the qualitative evidence is reported in tables separately but is integrated with the quantitative evidence later in the synthesis. During quantitative synthesis, a statistical meta‐analysis was considered but was not possible due to data heterogeneity. Therefore, a narrative synthesis was conducted following Popay et al.'s approach (Popay et al., 2006). Likewise, a meta‐synthesis was considered during qualitative synthesis, but data heterogeneity did not allow for this, so a narrative synthesis approach was adopted, which focused on common themes concerning positive and negative experiences related to HE and/or PA behaviour change within the interventions, and suggestions made by participants on how the intervention could be improved.

## RESULTS

The database searches identified 20,001 records. Following de‐duplication in EndNote, the application of the data restriction, and title and abstract screening, 268 studies were screened for eligibility at full text. Of the 268, 254 studies were excluded. Four studies were included from grey literature searches, and three studies from manually searching the reference lists of included studies. A total of 21 studies were included in the review (see Figure 1).

### Characteristics of included studies

The included studies comprised mixed methods (*n* = 6), quantitative (*n* = 4) and qualitative (*n* = 11) study designs. These were further categorized as evaluations (*n* = 7), feasibility studies (*n* = 4), exploratory studies (*n* = 8), a randomized controlled trial (*n* = 1) and a pilot study (*n* = 1). Studies were conducted in the United States (*n* = 7), Canada (*n* = 3), England (*n* = 3), the United Kingdom (*n* = 2), Northern Ireland (*n* = 1), Spain (*n* = 3), Ireland (*n* = 1) and Sweden (*n* = 1). Table 2 summarizes the characteristics of the included studies (Arevalo et al., 2023; Chauvenet et al., 2022; Frerichs et al., 2020; Gallagher et al., 2021; Hayton et al., 2019; Lai et al., 2019; Lawlor et al., 2019; Luft et al., 2023; Martin‐Hammond & Purnell, 2022; Moore et al., 2023; Quirk, 2024; Quirk & Haake, 2019, 2021; Ramji et al., 2022; Sanchez et al., 2021; Sanz‐Remacha et al., 2021, 2022, 2023; Springer et al., 2022; Wicklum et al., 2019, 2023).

**TABLE 2:** Study characteristics and intervention effectiveness.

Author(s)	Year	Location	Aims	Participant characteristics	Type of intervention, duration and setting	Study design and analysis	Intervention effectiveness: statistically significant effects only
Arevalo et al. (2023)	2023	The United States	To assess the feasibility of delivering a study protocol of a 3‐month community‐based intervention (CBI) and a home‐based intervention (HBI) to increase physical activity among low‐income, ethnic minority mothers	*N* = 30. Low‐income ethnic minority women. 30F	Physical activity; 3 months; local church	Mixed methods feasibility study; descriptive statistics and thematic analysis	Increase in objectively measured PA: significant increase in objectively measured PA among all participants PA outcomes: [Fitbit; *F*(1, 21) = 4.91, *p* = .038, $\eta_{p}^{2}$ = .19 (linear pattern)]. Cardiorespiratory fitness [VO_2_max; *F*(1, 25) = 7.84, *p* = .010, $\eta_{p}^{2}$ = .29 (linear pattern)]. Flexibility [Sit & Reach; *F*(1, 25) = 4.28, *p* =.049, $\eta_{p}^{2}$ = .15 (linear pattern)] from baseline to 3 months post‐intervention (*p* = .038) and differences between groups [Fitbit; *F*(1, 21) = 4.30, *p* = .051, $\eta_{p}^{2}$ = .17 (cubic pattern)] Increase in PA social support from family among all participants [*F*(1, 15) = 8.85, *p* = .009, $\eta_{p}^{2}$ = .37 (quadratic pattern)] Increased PA social support from friends: significant results among all participants [*F*(1, 15) = 4.35, *p* = .055, $\eta_{p}^{2}$ = .23 (cubic pattern)] Increase in PA self‐efficacy in the intervention compared to the control: significant differences between groups over time favouring the intervention [*F*(1, 18) = 6.28, *p* = .022, $\eta_{p}^{2}$ = .26 (linear pattern)] and [*F*(1,15) = 4.57, *p* = .049, $\eta_{p}^{2}$ = .23 (quadratic pattern)]
Chauvenet et al. (2022)	2022	USA	To understand how community garden participants experience the effects of participation on their fruit and vegetable consumption and health behaviours	*N* = 61. Rural, low income (majority female and African American). 12M; 49F	Healthy eating; duration not reported; community gardens	Qualitative exploratory study; thematic analysis	Not Applicable
Frerichs et al. (2020)	2020	USA	To evaluate the effects of the intensive intervention phase of Heart Matters on diet and physical activity behaviours, self‐efficacy and social support	*N* = 143 (*n* = 72 intervention, *n* = 71 delayed intervention control). Rural African Americans. 81F; 27M (completion) 23F; 12M (non‐completion)	Healthy eating and physical activity; 12 months; community faith‐based organization	Cluster randomized controlled trial (quantitative); descriptive statistics using mixed regression models	Increased HE self‐efficacy favouring the intervention: significant between‐group differences for salt intake (β = .31, 95% CI [.09, .53]) (*p* =.006) and dietary change adherence (β = .28, 95% CI [.02, .55]) (*p* = .036) Increases in HE social support favouring the intervention: for friends' encouragement (+4.45, 95% CI [1.3, 7.59]) (*p* = .0004) and family encouragement (+5.59, 95% CI [1.46, 9.73]) (*p* = .004) Increased self‐reported PA favouring the intervention: (OR = 2.86, 95% CI [1.18, 6.93]) (*p* = .02) Increased PA social support favouring the intervention: friend participation (+5.46, 95% CI [.84, 10.08]) (*p* = .0211), family participation (+7.25, 95% CI [2.00, 12.49]) (*p* = .0074)
Gallagher et al. (2021)	2021	Ireland	To assess the impact of the intervention on activity levels and assess the impact on self‐esteem measures, anxiety and depression scores, quality of life measures and physical parameters	*N* = 35. Individuals with serious mental illness. Gender not reported	Healthy eating and physical activity; 3 months; library and other community resources (not specified)	Quantitative feasibility study; pre and post descriptive statistical comparison	Increase in self‐reported PA within‐group over time: baseline minutes per week (*M* = 689, SD = 258), follow‐up minutes per week (Mean = 1575, SD = 486), *t*(34) = 12.79, (*p* <.001) Increased PA self‐esteem: significant within‐group: baseline Rosenberg scale score (*M* = 20.1, SD = 4.3) to follow‐up Rosenberg scale score (*M* = 23.2, SD = 3.4), *t*(34) = 3.14, *p* = .001 (*p* = .001)
Hayton et al. (2019)	2019	England	To examine how Cycling Projects mobilizes and deploy the variety of resources it requires to implement inclusive cycling programmes within the communities it operates	*N* = 47 (Interviews: *n* = 15. Focus group: *n* = 32). Individuals self‐identifying as low income, ethnic minority, living with a chronic health condition or low mental well‐being; informants^a^ (project stakeholders and facilitators). Gender not reported	Physical activity; duration not reported; community parks	Qualitative exploratory study; thematic analysis	Not Applicable
Lai et al. (2019)	2019	Canada	To evaluate an Indigenous‐led and community‐based health and wellness intervention in a remote and rural Indigenous community	*N* = 15. Rural Indigenous adults. 13F; 2M	Physical activity; 13 weeks; outside in the community or at a community setting (not specified)	Quantitative pre–post evaluation; descriptive statistics	Objectively measured PA within‐group: Increased frequency (times/week) of MVPA bouts >30 min within‐group over time: pre *M* (SE) = 2.4 ± .8; post *M* (SE) = 4.6 ± 1.1, *p* < .05 Increase in MVPA time (min/week) spent in bouts of >30 min within‐group over time: pre *M* (SE) = 73.6 ± 24.4; post *M* (SE) 139.1 ± 31.9, *p* < .05 Self‐reported PA: significant within‐group: Increase in MVPA time (min/week) over time within‐group: pre *M* (SE) = 174 ± 52; post *M* (SE) = 361 ± 79, *p* < .05
Lawlor et al. (2019)	2019	Northern Ireland	To test the operational aspects of the trial design in terms of recruitment, retention and outcome assessments. Ensure that the proposed methodological approach was feasible for a large‐scale trial. Gather participants' views on the acceptability of the intervention and trial design	*N* = 40 (26 interviewed in focus groups). Low SES older women. 40F	Physical activity; 3 months; community centres	Mixed methods feasibility study; descriptive statistics and framework analysis	Not Applicable
Luft et al. (2023)	2023	USA	To evaluate the effectiveness and implementation of mySTEPS, a heart‐healthy intervention adapted for women of low SES	Baseline = 44; Participants who started in Phase 1 = 42; Participants who completed Phase 2 = 19. Low SES women (majority Latina/African American). 44F	Healthy eating and physical activity; 4 months; community centres	Mixed methods process evaluation; descriptive statistics and thematic analysis	Self‐reported PA levels: significant within‐group difference for metabolic equivalents of ≥moderate intensity PA/week baseline (*M* = 48.82, SD = 47.59) to follow‐up (*M* = 85.16), representing a medium effect size (*d* = .73, 95% CI [.12, 1.33]), *p* = .03 Increased HE knowledge: significant within‐group increases from pre‐ (3.03) to post‐ (3.22) group education sessions (*t* = 2.13 (23), *p* < .05) Increased PA knowledge: significant within‐group increases from pre‐ (3.03) to post‐ (3.22) group education sessions (*t* = 2.13 (23), *p* < .05)
Martin‐Hammond and Purnell (2022)	2022	USA	To explore participants' experiences in an existing community‐based health education and behaviour change programme to reduce heart health risks in Black communities	*N* = 15. Black American & Informants (facilitators, directors, board members). Gender not reported	Healthy eating and physical activity (largely physical activity focused); 6 weeks; community setting and outside community spaces	Qualitative exploratory study; thematic analysis	Not Applicable
Moore et al. (2023)	2023	UK	To determine the feasibility of undertaking a community multicultural healthy eating education and cooking intervention featuring African‐Caribbean foods at a community organization by evaluating service users' and staff perceptions of the acceptability and relevance of using resources in real life/practice. To evaluate the potential impact of the intervention and resources on participants' food and cooking confidence, knowledge and behaviours	*N* = 32 (*n* = 22 participants, *n* = 10 informants). African Caribbean majority; low education majority. Participants: 17F; 4M; Other: 1. Informants: 10F	Healthy eating; 1 month; community centres	Mixed Methods feasibility study; descriptive statistics and thematic analysis	HE knowledge: increased significantly within‐group from pre (26%, *n* = 5) to post‐ (74%, *n* = 14) (*p* < .05) and follow‐up, FU (95%, *n* = 18) (*p* < .05)
Quirk and Haake (2019)	2019	England	To understand the perceptions of parkrun and the PROVE project for people living with long‐term conditions from the perspective of Parkrun volunteer Outreach Ambassadors	*N* = 15. Individuals with Long‐Term Conditions (LTC) & Informants (carers of someone with the LTC, specialist working in the field of the LTC). Gender not reported	Physical activity; duration not reported; community spaces and parks	Qualitative exploratory study; thematic analysis	Not Applicable
Quirk and Haake (2021)	2021	England	To understand the experience of delivering the parkrun PROVE project from the perspective of Outreach Ambassadors and the PROVE Project Manager, and provide guidance for organizations wanting to implement similar outreach initiatives	*N* = 11. Individuals with Long‐Term Conditions and Informants (Project Manager, Outreach Ambassadors). Gender not reported	Physical activity; duration not reported; community spaces and parks	Qualitative exploratory study; reflexive thematic analysis	Not Applicable
Quirk (2024)	2024	UK	To understand how community‐based initiatives like parkrun can reach underrepresented groups. To explore the perspectives of UK‐based parkrun Ambassadors to understand what Ambassadors have done to engage with underrepresented groups and their perspectives on what works, what does not work and why	*N* = 10. Informants in underserved areas (Outreach Ambassadors, Event Ambassadors and Regional Ambassadors). Gender not reported	Physical activity; duration not reported; community spaces and parks	Qualitative exploratory study; thematic analysis	Not Applicable
Sanchez et al. (2021)	2021	USA	To assess the feasibility of implementing a health promoter‐led Eat Healthy, Be Active intervention to improve obesity‐related behaviours among Hispanic women residing in a predominantly Hispanic rural community	*N* = 49. Rural Hispanic women. 49F	Healthy eating and physical activity; 6 weeks; variety of community settings	Quantitative pilot study; pre‐ and post‐ descriptive statistics	Self‐reported PA levels: significant within‐group difference in ‘engage in regular PA enough to sweat’ (*M* = 1.13, 95% CI: .84–1.42) to (mean = 1.83, 95% CI: 1.52–2.14), (*p* < .001) Increased HE knowledge within‐group: significant in nutrition label literacy (correctly identifying calorie content) increased significantly from pre‐intervention (25 of 49; 51.0%) to post‐intervention (38 of 49; 77.6%), *p* = .002 and (correctly identifying calorie intake increased from pre‐intervention (25 of 46; 51.0%) to post‐intervention (35 of 46; 76.1%), *p* = .043)
Sanz‐Remacha et al. (2021)	2021	Spain	To analyse women's perceptions of a set of motivational outcomes and affective consequences during and immediately after a PA intervention	*N* = 11. Low SES mothers (majority Roma ethnicity). 11F	Healthy eating and physical activity; 20 months community setting	Qualitative evaluation; thematic analysis	Not Applicable
Sanz‐Remacha et al. (2022)	2022	Spain	To describe the design and implementation of a two‐year multiple health behaviour change intervention. To assess the strengths and weaknesses of the intervention programme	*N* = 11. Low SES mothers (majority Roma ethnicity). 11F	Healthy eating and physical activity; 20 months; community setting	Qualitative exploratory study; thematic analysis	Not Applicable
Sanz‐Remacha et al. (2023)	2023	Spain	To analyse the first and second year follow‐ups of a community‐based healthy lifestyle programme on PA‐related variables, healthy eating and other health‐related outcomes in disadvantaged women, particularly among Roma women	*N* = 11. Low SES mothers (majority Roma ethnicity). 11F	Healthy eating and physical activity; 20 months; community setting	Qualitative evaluation; reflexive thematic analysis	Not Applicable
Springer et al. (2022)	2022	USA	To assess the amount of PA delivered via Stronger Austin's fitness classes when delivered in ‘real‐world’ community‐based settings. To explore highlights, perceived benefits and recommendations for enhancing the delivery of SA classes among participants and fitness class instructors	PA Engagement System for Observing Fitness Time (SOFIT) observations (*n* = 160); Focus group (*n* = 24); Open‐ended questionnaire (*n* = 258); Fitness Instructor personal interviews (*n* = 6). Low SES (majority Hispanic and African American) & Informants (instructors). Majority female, exact numbers not reported	Physical activity; duration not reported; community centres and parks	Mixed methods programme evaluation: descriptive statistics and thematic analysis	Not Applicable
Ramji et al. (2022)	2022	Sweden	To explore the impact of a CBPR‐informed physical activity intervention before and during the COVID‐19 pandemic from the perspective of women from a socially disadvantaged neighbourhood	*N* = 34. Arabic migrant women. 34F	Healthy eating and physical activity (largely physical activity focused); 3 months; community setting	Qualitative evaluation; pre‐ and post‐content analysis	Not Applicable
Wicklum et al. (2019)	2019	Canada	To evaluate the impact of the programme (improved PA levels, enhanced nutrition knowledge, increased social support and capacity for community building)	*N* = 49 (a total of 66 attended the programme for the first time, 26% of these attended fewer than three sessions and were excluded from analysis (*n* = 17)). Urban Indigenous women. 49F	Healthy eating and physical activity (largely physical activity focused); 2 months; community centre	Mixed methods evaluation; pre‐ and post‐descriptive statistics and thematic analysis	Objectively measured PA: Increased average weekly step count within‐group: week 2 (step counts were not collected in Week 1) (mean ± SD) 45,549 ± 20,227; week 8 (mean ± SD) 67,779 ± 21,360, *p* = .001 Increased confidence in activities for healthy living (PA group exercise): means and standard deviations not reported, authors report measures were taken pre and post programme, *p* = .042 Increased confidence in activities for healthy living (eating at least five servings of fruit and vegetables per day): means and standard deviations not reported, *p* = .002
Wicklum et al. (2023)	2023	Canada	To collate 3 years' worth of qualitative data between the first iteration of the programme and the second iteration and answer the question: ‘What does the (current) programme do?’	*N* = 31. Participants (*n* = 24); Facilitators (*n* = 4); Community champions (*n* = 3). Urban Indigenous women & Informants (facilitators and community champions). Gender not reported	Healthy eating and physical activity (largely physical activity focused); 2–3 months; community centre	Qualitative exploratory study; thematic analysis	Not Applicable

Abbreviations: F, female; M, male.

^a^ Individuals who are typically stakeholders or involved in the delivery of the intervention that provide supplementary or corroborative information about the underserved population based on their observations, interactions and knowledge.

### Characteristics of participants

#### Underserved populations

Characteristics of underserved populations were identified in all included studies, with most participants having multiple identities listed as part of the NIHR‐INCLUDE definition for underserved populations (NIHR, 2020). Fifteen studies reported either all or the majority of participants identified as an underserved ethnic minority in their country of residence (Arevalo et al., 2023; Chauvenet et al., 2022; Frerichs et al., 2020; Lai et al., 2019; Luft et al., 2023; Martin‐Hammond & Purnell, 2022; Moore et al., 2023; Ramji et al., 2022; Sanchez et al., 2021; Sanz‐Remacha et al., 2021, 2022, 2023; Springer et al., 2022; Wicklum et al., 2019, 2023). Ten studies reported on participants of low socio‐economic status (Arevalo et al., 2023; Chauvenet et al., 2022; Hayton et al., 2019; Lawlor et al., 2019; Luft et al., 2023; Quirk, 2024; Sanz‐Remacha et al., 2021, 2022, 2023; Springer et al., 2022), three on participants with long‐term conditions (e.g., dementia and learning disabilities) (Hayton et al., 2019; Quirk & Haake, 2019, 2021) and two with serious mental illness (Gallagher et al., 2021; Hayton et al., 2019). Gender information was provided in 14 of the 21 included studies, with all 14 studies including exclusively (Arevalo et al., 2023; Lawlor et al., 2019; Luft et al., 2023; Ramji et al., 2022; Sanchez et al., 2021; Sanz‐Remacha et al., 2021, 2022, 2023; Wicklum et al., 2019) or predominantly (80.3%–95.3%) (Chauvenet et al., 2022; Frerichs et al., 2020; Lai et al., 2019; Moore et al., 2023; Springer et al., 2022) female participants.

### What are the behaviour change characteristics, settings and modes of delivery of community‐centred healthy eating and physical activity approaches for underserved populations?

#### Type of intervention

Of the 21 included studies, 18 unique interventions were identified, as some studies reported on the same intervention but with different study designs and/or study aims (Quirk & Haake, 2019, 2021; Sanz‐Remacha et al., 2021, 2022, 2023; Wicklum et al., 2019, 2023). Of the 18 interventions, two were aimed at HE behaviours only (Chauvenet et al., 2022; Moore et al., 2023), seven at PA behaviours only (Arevalo et al., 2023; Hayton et al., 2019; Lai et al., 2019; Lawlor et al., 2019; Quirk, 2024; Quirk & Haake, 2019, 2021; Springer et al., 2022) and nine at both HE and PA behaviours (Frerichs et al., 2020; Gallagher et al., 2021; Luft et al., 2023; Martin‐Hammond & Purnell, 2022; Ramji et al., 2022; Sanchez et al., 2021; Sanz‐Remacha et al., 2021, 2022, 2023; Wicklum et al., 2019, 2023).

#### Intervention settings

All interventions were delivered in community settings within the VCSE sector, most notably community parks and outdoor spaces (*n* = 7) (Chauvenet et al., 2022; Hayton et al., 2019; Lai et al., 2019; Martin‐Hammond & Purnell, 2022; Quirk & Haake, 2019, 2021; Springer et al., 2022), community centres (*n* = 6) (Lawlor et al., 2019; Luft et al., 2023; Moore et al., 2023; Springer et al., 2022; Wicklum et al., 2019, 2023), faith‐based organizations (*n* = 2) (Arevalo et al., 2023; Frerichs et al., 2020) and libraries (*n* = 1) (Gallagher et al., 2021).

#### Intervention delivery

Interventions were predominantly group‐based, and intervention frequency varied considerably across the studies. All interventions were delivered entirely (Gallagher et al., 2021; Hayton et al., 2019; Lai et al., 2019; Martin‐Hammond & Purnell, 2022; Moore et al., 2023; Ramji et al., 2022; Sanchez et al., 2021; Sanz‐Remacha et al., 2021, 2022, 2023; Springer et al., 2022; Wicklum et al., 2019, 2023) or predominantly (Arevalo et al., 2023; Chauvenet et al., 2022; Frerichs et al., 2020; Lawlor et al., 2019; Luft et al., 2023; Quirk, 2024; Quirk & Haake, 2019, 2021) in person, with some interventions including elements that were accessed on an individual basis or online. These included one‐to‐one telephone support (Lawlor et al., 2019), social media applications (e.g., Facebook and Strava clubs) (Quirk & Haake, 2021; Wicklum et al., 2019) and written materials that participants took away to look at individually (e.g., leaflets and activity plans) (Arevalo et al., 2023; Lai et al., 2019; Lawlor et al., 2019; Martin‐Hammond & Purnell, 2022; Moore et al., 2023; Sanchez et al., 2021). Interventions were delivered by a range of stakeholders in diverse roles and professions, including *healthcare professionals* (e.g., student nurses, dieticians, nutritionists and kinesiologists) (Arevalo et al., 2023; Luft et al., 2023; Moore et al., 2023); *non‐healthcare professionals and volunteers*, such as lay health promoters and community centre staff (Hayton et al., 2019; Lawlor et al., 2019; Moore et al., 2023; Ramji et al., 2022; Sanchez et al., 2021); *intervention‐specific trained staff or volunteers from underserved populations*, including lay community members, project ambassadors and community champions (Chauvenet et al., 2022; Frerichs et al., 2020; Martin‐Hammond & Purnell, 2022; Quirk, 2024; Quirk & Haake, 2019, 2021; Wicklum et al., 2023) and *fitness instructors and exercise professionals* (Lai et al., 2019; Sanz‐Remacha et al., 2021, 2022, 2023; Springer et al., 2022; Wicklum et al., 2019).

#### Community‐centred approach

Partner organizations or individuals were commonly reported as a part of the intervention's community‐centred approach. This included faith‐based organizations (Arevalo et al., 2023; Frerichs et al., 2020; Martin‐Hammond & Purnell, 2022; Sanchez et al., 2021), community centres (Chauvenet et al., 2022; Gallagher et al., 2021; Lawlor et al., 2019; Moore et al., 2023; Springer et al., 2022), local government schemes (Chauvenet et al., 2022) and respected community leaders (e.g., Indigenous Elders) (Frerichs et al., 2020; Hayton et al., 2019; Lai et al., 2019; Lawlor et al., 2019; Moore et al., 2023; Quirk, 2024; Ramji et al., 2022; Sanz‐Remacha et al., 2021, 2022, 2023; Springer et al., 2022) who were trusted in their communities. Common non‐clinical strategies for promoting health and well‐being comprised promotion through socialization (Arevalo et al., 2023; Chauvenet et al., 2022; Frerichs et al., 2020; Gallagher et al., 2021; Lai et al., 2019; Lawlor et al., 2019; Luft et al., 2023; Martin‐Hammond & Purnell, 2022; Quirk, 2024; Quirk & Haake, 2021; Ramji et al., 2022; Sanz‐Remacha et al., 2021, 2022, 2023; Springer et al., 2022; Wicklum et al., 2019, 2023), inclusivity‐focused eligibility to the groups (Chauvenet et al., 2022; Ramji et al., 2022), health promotion delivered by relatable individuals with lived experience of local challenges (Frerichs et al., 2020; Martin‐Hammond & Purnell, 2022; Quirk, 2024; Quirk & Haake, 2019, 2021; Wicklum et al., 2019, 2023), familiar and accessible settings and assets (Frerichs et al., 2020; Gallagher et al., 2021; Hayton et al., 2019; Springer et al., 2022) and health promotion tailored to the cultural context of the participants (Lai et al., 2019; Martin‐Hammond & Purnell, 2022; Moore et al., 2023; Wicklum et al., 2019, 2023). Evidence of empowerment techniques to advocate individual control of participants' health was also evident. These included advocating choice and control tailored to participant capability during the intervention (Lai et al., 2019; Martin‐Hammond & Purnell, 2022; Quirk & Haake, 2019, 2021; Ramji et al., 2022); tailoring intervention content to individual norms, most notably socio‐cultural norms (Frerichs et al., 2020; Lai et al., 2019; Lawlor et al., 2019; Luft et al., 2023; Martin‐Hammond & Purnell, 2022; Moore et al., 2023; Quirk, 2024; Quirk & Haake, 2019, 2021; Sanchez et al., 2021; Sanz‐Remacha et al., 2021, 2022, 2023; Springer et al., 2022; Wicklum et al., 2019, 2023); low‐cost or free‐of‐charge resources (Chauvenet et al., 2022; Gallagher et al., 2021; Hayton et al., 2019; Luft et al., 2023; Ramji et al., 2022); encouraging participant reflection and autonomous learning through workshops and journals (Frerichs et al., 2020; Martin‐Hammond & Purnell, 2022; Sanchez et al., 2021; Sanz‐Remacha et al., 2021, 2022, 2023); interactive and practical skill building (e.g., cooking skills and cycling skills) (Arevalo et al., 2023; Hayton et al., 2019; Moore et al., 2023) and increasing participants' awareness of other opportunities to join HE and/or PA groups in their area beyond the intervention (Frerichs et al., 2020; Springer et al., 2022). These themes provide insight into how South et al.'s (2019) community‐centred approach criteria manifested in the included studies' HE and/or PA interventions.

#### Behaviour change techniques

Complete coding of BCTs according to the BCTO is provided in File S3, and BCT frequency mapping across all levels in each of the 18 interventions is provided in File S4. Inter‐rater reliability between the two BCT coders was 95.1% across the total number of BCTs available to code. After consensus discussions, 100% agreement was reached. Due to a low number of studies using comparator groups (*n* = 4) (Arevalo et al., 2023; Frerichs et al., 2020; Lawlor et al., 2019; Moore et al., 2023), and the majority of these not using a control group containing different BCTs from those used in the intervention group, BCTs in the comparator groups are not reported in the frequency mapping.

At least one BCT was identified in each of the 18 interventions. Level 1 BCT groupings are reported in Table 3. The most frequently reported Level 1 BCTs were social support (83%), guiding how to perform the behaviour (77%), monitoring behaviour (50%), goal‐directed behaviour (50%) and prompting focus on self‐identity (44%).

**TABLE 3:** BCTO Level 1 frequency table.

Level 1 entities	Interventions																		
	Int. 1 (Arevalo et al., 2023)	Int. 2 (Chauvenet et al., 2022)	Int. 3 (Frerichs et al., 2020)	Int. 4 (Gallagher et al., 2021)	Int. 5 (Hayton et al., 2019)	Int. 6 (Lai et al., 2019)	Int. 7 (Lawlor et al., 2019)	Int. 8 (Luft et al., 2023)	Int. 9 (Martin‐Hammond & Purnell, 2022)	Int. 10 (Moore et al., 2023)	Int. 11 (Quirk & Haake, 2019, 2021)	Int. 12 (Quirk, 2024)	Int. 13 (Sanchez et al., 2021)	Int. 14 (Sanz‐Remacha et al., 2021, 2022, 2023)	Int. 15 (Springer et al., 2022)	Int. 16 (Ramji et al., 2022)	Int. 17 (Wicklum et al., 2019)	Int. 18 (Wicklum et al., 2023)	Total Level 1 BCTs
Goal‐Directed BCT BCIO:007001^a^	✓		✓			✓	✓	✓	✓					✓			✓	✓	9
Monitoring BCT BCIO:007017	✓		✓			✓		✓	✓		✓			✓			✓	✓	9
Social Support BCT BCIO:007028	✓	✓	✓	✓		✓	✓	✓	✓		✓	✓		✓	✓	✓	✓	✓	15
Guide on how to perform behaviour BCT BCIO:007050	✓		✓	✓	✓	✓	✓	✓	✓	✓		✓	✓	✓	✓		✓		14
Suggest a different perspective on behaviour BCT BCIO:007302							✓	✓	✓		✓	✓							5
Increase awareness of behaviour BCT BCIO:007173				✓				✓	✓		✓	✓		✓		✓			7
Increase awareness of consequences BCT BCIO:007062	✓						✓		✓		✓			✓					5
Awareness of other people's thoughts feelings and actions BCT BCIO:007072				✓							✓			✓	✓	✓	✓		6
Associate learning BCT BCIO:007090									✓										1
Advise specific behaviour BCT BCIO:007168	✓			✓				✓				✓		✓					5
Manage mental processes BCT BCIO:007185								✓						✓					2
Prompt thinking related to successful performance BCT BCIO:007239							✓	✓	✓	✓				✓		✓			6
Advise how to change emotions BCT BCIO:007147	✓													✓					2
Restructure the environment BCT BCIO:007150			✓		✓						✓	✓		✓	✓				6
Prompt focus on self‐identity BCT BCIO:007157	✓		✓				✓		✓		✓			✓		✓		✓	8
Behavioural consequence BCT BCIO:007101														✓					1
Outcome consequence BCT BCIO:007186																✓			1
Total of Level 1 BCTs per intervention	8	1	6	5	2	4	7	9	10	2	8	6	1	14	4	6	5	4	

Abbreviations: BCT, behaviour change technique; Int, intervention.

^a^ Behaviour change intervention ontology identity number.

A meta‐regression on commonly used Level 1 BCTs on the estimated impact of each BCT on study outcomes was not possible due to heterogeneity and limited statistical significance among the studies. This made it difficult to reliably attribute individual BCTs to any intervention effects on HE/PA behaviours. However, as a visual inspection of patterns exercise, studies reporting statistically significant and positive change in HE (Luft et al., 2023; Moore et al., 2023; Sanchez et al., 2021; Wicklum et al., 2019) and PA (Arevalo et al., 2023; Frerichs et al., 2020; Gallagher et al., 2021; Lai et al., 2019; Luft et al., 2023; Sanchez et al., 2021; Wicklum et al., 2019) behaviours and the influence of these behaviours were found not to have an association with the number of Level 1 BCTs used in the interventions.

### What is the evidence that community‐centred HE and PA approaches are effective in improving eating and PA behaviour change outcomes for underserved populations?

#### Quantitative outcomes

Quantitative outcomes of the intervention were reported in 10 studies (Arevalo et al., 2023; Frerichs et al., 2020; Gallagher et al., 2021; Lai et al., 2019; Lawlor et al., 2019; Luft et al., 2023; Moore et al., 2023; Sanchez et al., 2021; Springer et al., 2022; Wicklum et al., 2019). Five reported outcomes related to HE behaviours (Frerichs et al., 2020; Luft et al., 2023; Moore et al., 2023; Sanchez et al., 2021; Wicklum et al., 2019), and four reported influences on HE behaviour change outcomes (e.g., self‐efficacy) (Frerichs et al., 2020; Luft et al., 2023; Moore et al., 2023; Sanchez et al., 2021). All studies reported within‐group differences and two between‐group differences (Frerichs et al., 2020; Moore et al., 2023), with one study having an intervention and a delayed intervention group (Frerichs et al., 2020) and the other reporting a sub‐group analysis according to ethnicity (Moore et al., 2023). Nine studies reported outcomes related to PA behaviours (Arevalo et al., 2023; Frerichs et al., 2020; Gallagher et al., 2021; Lai et al., 2019; Lawlor et al., 2019; Luft et al., 2023; Sanchez et al., 2021; Springer et al., 2022; Wicklum et al., 2019), and six reported influences on PA behaviour change outcomes (e.g., self‐esteem) (Arevalo et al., 2023; Frerichs et al., 2020; Gallagher et al., 2021; Lawlor et al., 2019; Luft et al., 2023; Wicklum et al., 2019). All studies reported within‐group and three between‐group (Arevalo et al., 2023; Frerichs et al., 2020; Lawlor et al., 2019), where two studies had an intervention and a delayed intervention group (Frerichs et al., 2020; Lawlor et al., 2019), and one study compared a community‐based PA intervention with a home‐based PA intervention (Arevalo et al., 2023).

#### Healthy eating behaviour change quantitative outcomes

Indicators of HE behaviour change were measured by: *frequency of food intake* (Frerichs et al., 2020; Luft et al., 2023), *dietary guideline compliance* (Moore et al., 2023), *nutrition goal achievement* (Wicklum et al., 2019), *consumption quantity measuring* (Sanchez et al., 2021; Wicklum et al., 2019), *attitudes to HE practices* (Moore et al., 2023) and *consumption confidence* (Wicklum et al., 2019). All HE behaviour outcomes were self‐reported and measured using various data collection methods, including questionnaires (Frerichs et al., 2020; Luft et al., 2023; Moore et al., 2023; Sanchez et al., 2021), Likert scales (Moore et al., 2023; Wicklum et al., 2019) and frameworks (Wicklum et al., 2019), with only one study using a validated outcome measure, the dietary FLASHE (Family Life, Activity, Sun, Health and Eating) screener (Frerichs et al., 2020).

Dietary behaviours measuring changes to *frequency of food intake* were reported as not significant within or between groups (Frerichs et al., 2020). In terms of *compliance with HE guidance* and *attitudes to HE practices*, studies reported a small percentage increase from pre‐ to post‐intervention within groups, which were statistically significant and were not affected by ethnicity (Moore et al., 2023). Regarding *consumption quantity* within groups, one study reported mixed results with no clear evidence of increased or decreased quantity as a result of the intervention (Sanchez et al., 2021), whilst another study reported increased fruit and vegetable consumption (Wicklum et al., 2019). Regarding fruit and vegetable *consumption confidence*, one study reported small significant improvements (Wicklum et al., 2019) and reported that approximately half of the participants (53%) had significantly increased confidence in eating at least five servings of fruits and vegetables per day (Wicklum et al., 2019).

Influences on HE behaviours included: *self‐efficacy* (Frerichs et al., 2020), *social support* (Frerichs et al., 2020), *perceived stress* (Luft et al., 2023; Sanchez et al., 2021), *HE knowledge* (Luft et al., 2023; Moore et al., 2023; Sanchez et al., 2021), *intervention acceptability* (Luft et al., 2023; Moore et al., 2023), *recipe and cooking skills* (Moore et al., 2023) and *eating out in restaurants* (Sanchez et al., 2021). All influences were self‐reported and measured using various data collection methods such as questionnaires (Luft et al., 2023; Moore et al., 2023; Sanchez et al., 2021), Likert scales (Frerichs et al., 2020; Moore et al., 2023) and other scales (Luft et al., 2023), with one study using two validated outcome measures, the 6‐item Healthcare Climate questionnaire and the Short Form Perceived Stress Scale (PSS) (Luft et al., 2023).

Three studies found evidence of the intervention having significant positive within‐group change in participant *HE knowledge* (Luft et al., 2023; Moore et al., 2023; Sanchez et al., 2021), with no significant difference in increased HE knowledge between ethnicity groups post‐intervention (Moore et al., 2023). *Intervention acceptability and delivery* were reported as significantly increased within groups in two studies (Luft et al., 2023; Moore et al., 2023), with no significant differences found between groups post‐intervention in one study (Moore et al., 2023). Small decreases in *perceived stress* within groups were observed in one study (Luft et al., 2023) and no effect in another (Sanchez et al., 2021). *Self‐efficacy* differences were not reported within groups, but significant positive effects between groups, favouring the intervention group compared to the delayed intervention group, were observed (Frerichs et al., 2020). One study reported significant positive and negative effects in *social support* within and between groups, as participants experienced both encouragement and discouragement from their friends and families (Frerichs et al., 2020). Positive improvements in *cooking skills* were observed within groups and in the sub‐group analysis (Moore et al., 2023). No significant effects were found within groups regarding *the frequency of eating out in restaurants* (Sanchez et al., 2021).

In sum, intervention effects on measures of HE behaviour change were either modest or not statistically significant. However, overall studies reported positive effects on the influences of HE behaviours, rather than on HE behaviour change itself.

#### Physical activity behaviour change quantitative outcomes

Indicators of PA behaviour change were measured by: *objective levels of PA* (Arevalo et al., 2023; Lai et al., 2019; Lawlor et al., 2019; Springer et al., 2022; Wicklum et al., 2019), including tracking technology (Arevalo et al., 2023; Lawlor et al., 2019; Wicklum et al., 2019) and objective levels of vigorous PA (Lai et al., 2019; Springer et al., 2022); *self‐reported levels of PA* (Arevalo et al., 2023; Frerichs et al., 2020; Gallagher et al., 2021; Lai et al., 2019; Luft et al., 2023; Sanchez et al., 2021), including four studies that specifically reported on self‐reported levels of moderate‐vigorous PA (Frerichs et al., 2020; Lai et al., 2019; Luft et al., 2023; Sanchez et al., 2021); *percentage of participants meeting PA recommendations* (Arevalo et al., 2023) and the number of participants *meeting their PA goals* (Wicklum et al., 2019). These were measured using various data collection methods, including tracking technology (Arevalo et al., 2023; Lai et al., 2019; Lawlor et al., 2019; Wicklum et al., 2019), questionnaires (Arevalo et al., 2023; Frerichs et al., 2020; Gallagher et al., 2021; Lai et al., 2019; Sanchez et al., 2021), recall interviews (Luft et al., 2023), Likert scales (Sanchez et al., 2021), structured observations (Springer et al., 2022) and frameworks (Wicklum et al., 2019). Validated outcome measures were used in five studies. They included the Check and Line Questionnaire (Arevalo et al., 2023), the EPIC‐Norfolk PA Questionnaire (Epaq2) (Gallagher et al., 2021), the Godin‐Shephard Leisure Time Activity Questionnaire (Lai et al., 2019), the 7‐Day PA Recall Interview (PAR) (Luft et al., 2023) and the SOFIT (Structured Observation of Fitness Instruction Time) tool (Springer et al., 2022).

Four studies found evidence of significant positive within‐group effects in *objectively measured PA* (Arevalo et al., 2023; Lai et al., 2019; Springer et al., 2022; Wicklum et al., 2019), with one study finding objectively measured PA significantly higher in the community group compared to the home‐based group (Arevalo et al., 2023). One study measured objective PA but did not report between‐group differences in PA (Lawlor et al., 2019). Four studies found significant positive within‐group effects on *self‐reported PA levels* (Frerichs et al., 2020; Gallagher et al., 2021; Lai et al., 2019; Luft et al., 2023), one study found small within‐group effects (Sanchez et al., 2021), but another found no significant change (Arevalo et al., 2023). Self‐reported PA levels between groups were reported as not significant in one study (Arevalo et al., 2023) but positive change in another (Frerichs et al., 2020). One study reported on the *percentage of participants meeting PA recommendations*, which was over half of the participants post‐intervention. However, this was not measured at baseline, so intervention effects cannot be determined (Arevalo et al., 2023). One study found evidence of a small positive change in *achievement of PA goals*, with over half of participants indicating their PA goals were achieved during the programme (Wicklum et al., 2019).

Influences on PA behaviours included: *self‐efficacy* (Arevalo et al., 2023; Frerichs et al., 2020), *social support* (Arevalo et al., 2023; Frerichs et al., 2020), *self‐esteem* (Gallagher et al., 2021), *quality of life* (Gallagher et al., 2021), *anxiety and depression* (Gallagher et al., 2021; Lawlor et al., 2019), *intervention acceptability* (Lawlor et al., 2019; Luft et al., 2023), *perceived stress* (Luft et al., 2023), *PA knowledge* (Luft et al., 2023) and *reasons for not engaging in PA* (Wicklum et al., 2019). Influences were measured using various data collection methods, including Likert scales (Frerichs et al., 2020; Wicklum et al., 2019), other scales (Arevalo et al., 2023; Gallagher et al., 2021; Lawlor et al., 2019; Luft et al., 2023) and questionnaires (Gallagher et al., 2021; Lawlor et al., 2019; Luft et al., 2023). Four studies used validated measures, which included: the Barriers Self‐Efficacy Scale (BARSE) (Arevalo et al., 2023), the Self‐Efficacy for Physical Performance Scale (Arevalo et al., 2023), Rosenberg Self‐Esteem Scale (RSE) (Gallagher et al., 2021), EuroQol 5 Dimension (EQ‐5D) (Gallagher et al., 2021), Hospital Anxiety and Depression Scale (HADS) (Gallagher et al., 2021; Lawlor et al., 2019), 6‐item version of the Health Care Climate Questionnaire (HCCQ) (Luft et al., 2023) and the Perceived Stress Scale (PSS) (Luft et al., 2023).

In terms of *self‐efficacy* improvement, no significant effects were found within groups (Arevalo et al., 2023; Frerichs et al., 2020). One of the studies reported no significant difference between groups (Frerichs et al., 2020), and the other reported significant differences between groups favouring the community‐based group over the home‐based group (Arevalo et al., 2023). Regarding *social support* within groups, two studies found significant improvements (Arevalo et al., 2023; Frerichs et al., 2020), with one study reporting significant between‐group differences (Frerichs et al., 2020) and the other reporting no differences (Arevalo et al., 2023). One study reported significant increases in *self‐esteem* within groups, but no within‐group change in quality of life (Gallagher et al., 2021). *Anxiety and depression* were measured in two studies, with one study reporting no significant difference within the group (Gallagher et al., 2021) and the other not reporting differences within or between groups (Lawlor et al., 2019). Likewise, *intervention acceptability* was measured but not reported in one study (Lawlor et al., 2019) and reported as high post‐intervention in another study (Luft et al., 2023). One study reported small decreases in *perceived stress* within groups and small increases in *PA knowledge* within groups (Luft et al., 2023). Specific *reasons for not engaging in PA* were given by over half of participants in one study (Wicklum et al., 2019).

### What are the experiences of underserved populations who engage in behaviour change community‐centred eating and PA approaches?

#### Qualitative findings

Qualitative findings were reported in 17 studies (Arevalo et al., 2023; Chauvenet et al., 2022; Hayton et al., 2019; Lawlor et al., 2019; Luft et al., 2023; Martin‐Hammond & Purnell, 2022; Moore et al., 2023; Quirk, 2024; Quirk & Haake, 2019, 2021; Ramji et al., 2022; Sanz‐Remacha et al., 2021, 2022, 2023; Springer et al., 2022; Wicklum et al., 2019, 2023). Of these, 10 studies reported on HE behaviours (Chauvenet et al., 2022; Luft et al., 2023; Martin‐Hammond & Purnell, 2022; Moore et al., 2023; Ramji et al., 2022; Sanz‐Remacha et al., 2021, 2022, 2023; Wicklum et al., 2019, 2023), and 15 on PA behaviours (Arevalo et al., 2023; Hayton et al., 2019; Lawlor et al., 2019; Luft et al., 2023; Martin‐Hammond & Purnell, 2022; Quirk, 2024; Quirk & Haake, 2019, 2021; Ramji et al., 2022; Sanz‐Remacha et al., 2021, 2022, 2023; Springer et al., 2022; Wicklum et al., 2019, 2023). Table 4 summarizes the positive and negative experiences reported by participants engaging in HE community‐centred behaviour change approaches and suggestions on how the interventions could be improved. Table 5 reports the same data for PA behaviours.

**TABLE 4:** Healthy eating positive/negative themes and subthemes and suggestions to improve the intervention.

	Themes	Subthemes
Positive outcomes	Knowledge & empowerment (Chauvenet et al., 2022; Moore et al., 2023; Ramji et al., 2022; Sanz‐Remacha et al., 2021, 2022, 2023; Wicklum et al., 2019, 2023)	Increased focus Participants felt like role models Increased access to healthy foods Increased healthy eating knowledge and awareness No‐cost intervention increased accessibility Participants felt more confident Person/people who delivered the intervention instilled confidence in participants
	Social & community (Chauvenet et al., 2022; Martin‐Hammond & Purnell, 2022; Ramji et al., 2022; Sanz‐Remacha et al., 2021, 2023; Wicklum et al., 2019, 2023)	Increased community cohesion Increased socialization Increased social support Positive impact on families of participants Social environment enhanced motivation
	Practical support & resources (Martin‐Hammond & Purnell, 2022; Wicklum et al., 2019)	Increased access to other local healthy eating resources
	Intervention suitability (Chauvenet et al., 2022; Martin‐Hammond & Purnell, 2022; Ramji et al., 2022; Sanz‐Remacha et al., 2021, 2022; Wicklum et al., 2019, 2023)	Intervention made participants feel connected to cultural traditions Participants were satisfied with the intervention Participants liked that the intervention took place in a safe, familiar environment Intervention was tailored to the participants Intervention was flexible (e.g., length, location, content and childcare) Person/people who delivered the intervention were relatable (e.g., being able to identify with them or their context)
	Noticeable changes in healthy eating behaviours (Sanz‐Remacha et al., 2021, 2023; Wicklum et al., 2023)	Increased healthy food purchasing Participants set goals and noticed they could achieve them Incorporated healthy eating habits into daily lives
	Well‐being (Chauvenet et al., 2022; Moore et al., 2023; Sanz‐Remacha et al., 2021; Wicklum et al., 2023)	Positive effect on mental health of participants Increased enjoyment and overall well‐being
Negative outcomes	Lack of support or motivation (Martin‐Hammond & Purnell, 2022; Ramji et al., 2022; Sanz‐Remacha et al., 2023; Wicklum et al., 2019)	Lack of support from family or friends Lack of support from the wider community Not motivated to change healthy eating behaviours, initial refusal of intervention Low motivation Resistance to change in eating habits
	Conflicting responsibilities (Ramji et al., 2022; Sanz‐Remacha et al., 2022; Wicklum et al., 2019)	Participants are time poor Caring responsibilities (e.g., childcare) Conflicting work schedules Participants feel guilty that the intervention is taking them away from their responsibilities
	Intervention limitations or unsuitability (Martin‐Hammond & Purnell, 2022; Moore et al., 2023; Sanz‐Remacha et al., 2022; Wicklum et al., 2019)	Person/people who delivered the intervention are not relatable Logistical barriers to getting to the intervention Food ingredients recommended in the intervention are too expensive for the participants Intervention is too tightly scheduled Not enough people delivering the intervention
	Participant inter‐group issues (Chauvenet et al., 2022; Sanz‐Remacha et al., 2021, 2022; Wicklum et al., 2023)	Resentment of other participants Breakdown in group cohesion
	Healthcare issues (Wicklum et al., 2019)	Personal health issues preventing success (e.g., stress, post‐partum depression and long‐term conditions) Mistrust in the healthcare system
Suggestions to improve HE interventions	More practical (Moore et al., 2023; Wicklum et al., 2019)	Cooking advice Recipes
	More information on different diets (Moore et al., 2023)	Vegan diet information
	More individualized approaches (Moore et al., 2023; Sanz‐Remacha et al., 2021, 2022)	Promote HE choices rather than strict guidelines
	Prioritizing group activities (Ramji et al., 2022; Wicklum et al., 2019)	
	Increasing attendance options (Sanz‐Remacha et al., 2022)	
	More time allocated for participants to interact with experts who deliver the intervention (Wicklum et al., 2019)	
	Provide social networks outside of group meeting times (Wicklum et al., 2019)	Support network that goes beyond the allocated session times
	Recognize that HE behaviour change is harder than PA behaviour change (Sanz‐Remacha et al., 2023)	Adapt the intervention's content to emphasize the difficulty of HE behaviour change compared to PA behaviour change

**TABLE 5:** Physical activity: positive/negative themes and subthemes and suggestions to improve the intervention.

	Themes	Subthemes
Positive outcomes	Empowerment (Arevalo et al., 2023; Lawlor et al., 2019; Martin‐Hammond & Purnell, 2022; Quirk, 2024; Quirk & Haake, 2021; Ramji et al., 2022; Sanz‐Remacha et al., 2021, 2022, 2023; Wicklum et al., 2019, 2023)	Increased self‐confidence Increased access to other physical activity resources Positive learning experience Increased awareness, opened doors and broke down physical activity myths Increased physical activity knowledge Greater autonomy Participants felt like role models Increased motivation
	Support (Arevalo et al., 2023; Martin‐Hammond & Purnell, 2022; Sanz‐Remacha et al., 2021; Wicklum et al., 2019)	Social support Participants felt solidarity with the other women participating
	Socialization and community cohesion (Martin‐Hammond & Purnell, 2022; Ramji et al., 2022; Springer et al., 2022; Wicklum et al., 2019)	Increased connection with peers Valued diverse participants within the classes Community building Valued multigenerational participants
	Perceived health benefits (Arevalo et al., 2023; Ramji et al., 2022; Sanz‐Remacha et al., 2021, 2023; Springer et al., 2022; Wicklum et al., 2023)	Improved stress, sleep and mood Increased mental health and well‐being Reduced frustration Relieved pain
	Intervention suitability (Hayton et al., 2019; Lawlor et al., 2019; Luft et al., 2023; Martin‐Hammond & Purnell, 2022; Quirk & Haake, 2019; Ramji et al., 2022; Sanz‐Remacha et al., 2021, 2022; Springer et al., 2022; Wicklum et al., 2019, 2023)	Intervention was inclusive and accessible Participants were satisfied with the intervention Participants liked that the intervention took place in a safe, familiar environment Cost‐effective, volunteer‐led intervention preferred Participants liked that social media used for engagement Intervention was flexible (e.g., length, location, content and childcare) Person/people who delivered the intervention were relatable, trusted and skilled Participants liked the historical and cultural context of the intervention Participants found the intervention accountability structures helpful (e.g., monitoring behaviour)
	Noticeable positive changes in PA behaviour (Ramji et al., 2022; Sanz‐Remacha et al., 2023; Wicklum et al., 2019)	Improved motor skills, physical fitness and step counts Anecdotal evidence from families noticing their family members' PA behaviour had changed
Negative outcomes	Lack of support, motivation or knowledge (Arevalo et al., 2023; Martin‐Hammond & Purnell, 2022; Ramji et al., 2022; Sanz‐Remacha et al., 2021; Wicklum et al., 2019)	Low participant self‐confidence to complete physical activity Lack of support from participants' families Lack of knowledge or awareness of benefits of physical activity Participants were not motivated to change physical activity habits, initial refusal of intervention Low motivation influenced by stress Telephone support was not helpful
	Conflicting responsibilities (Arevalo et al., 2023; Ramji et al., 2022; Sanz‐Remacha et al., 2022; Wicklum et al., 2019)	Caring and household responsibilities Conflicting work schedules Participants felt guilty that the intervention is taking them away from their responsibilities
	Intervention limitations or unsuitability (Hayton et al., 2019; Martin‐Hammond & Purnell, 2022; Quirk, 2024; Quirk & Haake, 2019, 2021; Sanz‐Remacha et al., 2022)	Lack of people delivering the intervention Intervention needs a more autonomous model Overreliance on volunteers to deliver the intervention Limited communication channels to reach those who could benefit from the intervention Difficulty demonstrating intervention impact Logistical challenges (e.g., tight programme schedule) The person/people who delivered the intervention were not relatable to the participants
	Wider contextual barriers (Lawlor et al., 2019; Quirk, 2024; Wicklum et al., 2019)	Wider inequity in society High cost of physical activity (e.g., memberships and equipment) Participants felt unsafe doing physical activity outside alone
	Healthcare issues (Wicklum et al., 2019)	Personal health issues preventing success (e.g., stress, post‐partum depression and long‐term conditions) Mistrust in the healthcare system
Suggestions to improve PA interventions	Increase support structures and motivation (Arevalo et al., 2023; Hayton et al., 2019; Quirk, 2024; Quirk & Haake, 2021; Ramji et al., 2022; Springer et al., 2022; Wicklum et al., 2019)	Keep interventions face to face Group‐based preferred Offer peer support Be realistic as regards the pace of participants' behaviour change by making interventions longer Support change through competition or rewards‐based goals
	Improve intervention delivery and outreach (Hayton et al., 2019; Martin‐Hammond & Purnell, 2022; Quirk & Haake, 2019, 2021; Sanz‐Remacha et al., 2021, 2022; Springer et al., 2022; Wicklum et al., 2019)	Offer more flexible, varied activities Tailor outreach to the underserved groups? Lower the barriers to entry Provide childcare facilities Use technology to improve intervention content and maintain social networks More time with experts Make sure the person delivering the intervention is appropriate
	Use healthcare professionals and stakeholder networks to prescribe or signpost participants to the intervention (Quirk & Haake, 2019)	

### Integration of the quantitative and qualitative evidence: summary of findings

#### Healthy eating evidence

This review found preliminary evidence, both quantitative and qualitative, that interventions using community‐centred approaches can improve HE outcomes for underserved populations. Whilst intervention effects on HE behaviour change were either significant with modest changes or not statistically significant, participants qualitatively reported noticeable changes that suggested some behaviour change. Significant improvements in factors that influence HE behaviours, such as nutrition knowledge and skills, were found, as shown in both quantitative and qualitative data. Intervention effects on social support were mixed, with the evidence identifying both encouraging and discouraging factors. Despite general agreement between the quantitative and qualitative data, improved empowerment related to HE emerged as a major qualitative theme but was not widely measured quantitatively and showed little improvement when assessed. In sum, the improvements were more pronounced in outcomes related to participants' beliefs about their ability to change their eating behaviour, rather than in measurable behaviour change.

Both quantitative and qualitative evidence found that the experiences of underserved populations engaging in community‐centred HE interventions were heavily influenced by the perceived suitability of the intervention for the population. The delivery source (the facilitator who delivered the intervention first‐hand) was noted particularly in the qualitative evidence to positively affect experiences. Participants identified more time with relatable facilitators as a frequent recommendation to improve the interventions. Both quantitative and qualitative data suggested high acceptability of the interventions, with positive experiences linked to increased well‐being and social support reported in the qualitative data and, in limited cases, the quantitative data.

#### Physical activity evidence

Overall improvements in PA behaviour change were noted in both data sets; however, disparities were found between the quantitative and qualitative evidence in terms of how behaviour change was reported. The quantitative data showed some improvements in objective and self‐reported PA, with some studies reporting over half of the participants meeting recommendations or their PA goals post‐intervention. However, the qualitative data had limited experiential reports of improved fitness and step counts, suggesting that interview topic guides did not include questions on these subjects. Instead, the qualitative data more often reported improvements in other areas of health, such as well‐being, socialization and empowerment, which showed limited change in the quantitative data.

The experiences of underserved populations engaging in community‐centred PA interventions were unclear from the quantitative data, as intervention acceptability was measured in very few studies. However, positive and negative experiences in the qualitative data were heavily influenced by the intervention's suitability for the population and how easily participation could fit into the participants' lives in terms of accessibility, levels of motivation and conflicting responsibilities. The context and role of the person delivering the intervention were also reported as important factors in positive experiences.

## DISCUSSION

This review captured the characteristics of 18 HE and/or PA community‐centred interventions, within 21 studies, for underserved populations and assessed the studies' intervention effectiveness and participant experiences of the intervention.

A previous review on community HE/PA interventions for underserved populations failed to report community‐centred approach characteristics (Everson‐Hock et al., 2013). Results from this study address this gap and found community‐centred approaches in HE/PA interventions for underserved populations involved local partners, relatable facilitators and culturally tailored content delivered in accessible settings. Empowerment strategies included supporting participant choice, practical skill building, reflection and free resources. These community‐centred approaches provided the context for the BCTs used in the HE/PA interventions and were reported as a key factor contributing to intervention acceptability and suitability. This novel insight is pertinent to developing future HE/PA community‐centred interventions for underserved populations.

Consistent with the findings of this review, previous reviews found that goal‐directed, self‐monitoring and guiding how to perform behaviours are common BCT strategies in HE/PA interventions that target underserved populations (e.g., low SES, older adults and obese adults) (Bull et al., 2018; French et al., 2014; Froome et al., 2023; Samdal et al., 2017). However, this review also highlighted the consistent inclusion of social support BCTs across interventions, suggesting their perceived importance among programme designers when adopting a community‐centred approach to intervention implementation. Furthermore, significant improvements in social support in both quantitative and qualitative aspects, particularly in the PA data, highlight its practical effectiveness in influencing behaviour change. These findings suggest that fostering social networks by using a community‐centred approach that emphasizes socialization can enhance motivation and adherence to PA in underserved contexts.

This mixed methods review identified 18 interventions within 21 studies that demonstrated some HE and PA interventions were effective in changing behaviour, although measurable effects related to behaviour change were low. Improvements were largely related to influences on behaviour change rather than direct changes in HE/PA behaviours themselves. This may be due to the wider social determinants of health, where the physical or social environment could have stronger effects on behaviour change, given the target population. This finding is consistent with a study on the effects of a behaviour change intervention on HE and PA levels in women that found the intervention did not improve HE or PA levels but did affect intermediate factors such as control and self‐efficacy (Baird et al., 2014). Therefore, HE/PA behaviour change interventions potentially exert a protective effect on psychological behavioural factors such as social support and self‐efficacy in the short term, which may have provided participants with helpful strategies that could be beneficial for behaviour change in the future, but did not have a direct impact on behaviour change within the intervention research period.

Intervention length may have also contributed to the low impact and small measurable behaviour change effects, with most interventions, where duration was reported, spanning 3 months or less (84%). This is arguably too short a timeframe for behaviour change to occur, with evidence suggesting maintenance of HE and PA behaviour change requires interventions that are over 24 weeks in length (Fjeldsoe et al., 2011). The literature further suggests that contextually relevant behaviour change interventions, which this review has highlighted as important for positive outcomes in underserved populations, require an increased focus on longer‐term behavioural change (Baird et al., 2014; Cash et al., 2023).

The qualitative evidence in this review offers practical insights for future HE and/or PA community‐centred interventions aimed at underserved populations. Behaviour‐change‐related outcomes regarding participants' beliefs and experiences of the intervention were largely positive, with the context and role of the facilitator in supporting behaviour change and the intervention's suitability for the population (e.g., accessibility and relatability) cited as common reasons for positive experiences. Negative experiences and suggestions for intervention improvement largely concurred with the importance of these themes, with intervention unsuitability and contextual participant issues (e.g., health, conflicting responsibilities and inter‐group disagreement) commonly cited as reasons for intervention disengagement. This concurs with previous qualitative findings, where common facilitators and barriers include the intervention source delivering the interventions having appropriate knowledge and understanding of the population, their ability to address misunderstood beliefs about HE and PA, and intervention developers tailoring implementation strategies from the outset (Cooper et al., 2021; Everson‐Hock et al., 2013; Johnson et al., 2011).

### Implications for future research, community practice, policymakers and intervention developers

In the absence of substantial evidence regarding intervention effectiveness in the current literature, intervention developers, community practice, policymakers and researchers of future HE and/or PA community‐centred interventions should consider the findings of the qualitative evidence in this review useful in intervention development and implementation practice. This includes tailoring interventions to the population's cultural context early in development phases, with particular attention to embedding these considerations within intervention delivery modes from the outset. Additionally, this review has demonstrated that the facilitator (the person who delivers the intervention firsthand) is a cornerstone figure in using a community‐centred approach and intervention acceptability. Facilitators are often overlooked in evaluation research (McKeon et al., 2021). Future research should investigate the facilitator role further, including its bearing on positive participant outcomes, to understand how HE/PA interventions can be optimized for underserved populations.

To address the limited evidence of effectiveness in these interventions, future reviews should consider methodological limitations that may be less affected by data heterogeneity and yield more conclusive results through subgrouping analyses by intervention delivery mode or narrowing study inclusion criteria to specific groups within underserved populations (e.g., ethnicity and education) (Popay et al., 2006). However, this would need to be weighed up against the risk of losing intersectional identities within the samples.

Community practice policymakers should also consider how the use of community‐centred approaches contributes to intervention effectiveness, engagement and use specific strategies (e.g., local partners, relatable facilitators, empowerment strategies and culturally tailored content delivered in accessible settings) that this review found to facilitate positive participant experiences of the interventions in underserved populations. Furthermore, public health policy needs better tools to consider how to influence the wider determinants of health, including the impact of the physical and social environment (e.g., using a community‐centred approach), with many community‐oriented interventions (e.g., social prescribing) highlighting that there are substantial data gaps in this area (Khan et al., 2023). Without these considerations, interventions may struggle to achieve behaviour change in underserved populations.

Furthermore, policymakers and future researchers should consider implementing longer intervention periods when targeting behaviour change in underserved populations, which are more likely to build and reinforce behaviour change. Future studies, including randomized controlled trials, would also benefit from exploring the relationship between the application of BCTs within community HE/PA interventions and their relation to effective behaviour change in underserved populations (Bull et al., 2018), including applications of more statistical methods (e.g., a meta‐regression).

### Strengths and limitations

A strength of this review is its pioneering use of the BCTO for evidence synthesis. Employing the BCTO enabled the research team to identify intervention characteristics more systematically and comprehensively than other BCT‐identifying tools. Another strength is this review's use of the recently coined NIHR‐INCLUDE definition of underserved populations, enabling intersectionality of identity to be accounted for within study populations. Additionally, the use of South et al.'s (2019) definition of community‐centred approach to screen and analyse the interventions, rather than generalizing them to only community settings, highlighted how these interventions leverage community‐centred methods, assets and knowledge to target HE and PA behaviour change in underserved populations. Finally, by analysing both the quantitative and qualitative evidence in a single convergent segregated synthesis, this review robustly identifies research gaps and directions for future investigations in the field.

Regarding limitations, despite its strengths, BCT coding using the BCTO was a time‐consuming process. Additionally, as the tool remains largely untested in evidence synthesis, it requires some interpretation of the user guidelines. Furthermore, caution is warranted when comparing the frequency of BCTs across interventions, given the differences in target populations, settings, community‐centred strategies employed and delivery modes. Additionally, many of the included interventions lacked explicit descriptions of BCTs within their descriptions and processes, and behaviour change outcomes were largely self‐reported. A further limitation was that the country inclusion criteria for this review were narrow to manage scope and did not account for varying health systems or different characteristics of underserved populations between countries.

## CONCLUSION

This is the first review to synthesize the quantitative and qualitative evidence (effectiveness and experience) on community‐centred behaviour change interventions for HE and/or PA for underserved populations. The review also identifies common characteristics of HE and/or PA community‐centred behaviour change interventions for underserved populations, using the BCTO, and highlights the following shared BCTs as most commonly used: social support, guiding how to perform behaviour, monitoring, goal‐directed behaviour and prompting focus on participant self‐identity. However, the evidence on the effectiveness of these interventions in changing HE/PA behaviours in underserved populations remains limited, with more substantial evidence for improved outcomes related to the influences on HE/PA behaviour change (e.g., social support and self‐efficacy). Qualitative evidence suggests community‐centred approaches hold promise for improving HE/PA behaviours, particularly when the intervention includes tailoring to participants' lives and the intervention is delivered by a relatable, knowledgeable source. Further research is needed to assess the long‐term impacts on behaviour change, investigate the contextual mechanisms used to tailor behaviour change interventions for underserved populations, and explore the relationship between intervention source and delivery of HE/PA behaviour change interventions targeted at underserved populations.

## AUTHOR CONTRIBUTIONS

**Jessica Marshall:** Conceptualization; investigation; writing – original draft; methodology; validation; visualization; writing – review and editing; software; formal analysis; project administration; data curation; resources. **Anne‐Marie Minihane:** Conceptualization; funding acquisition; methodology; supervision; writing – review and editing. **Stephanie T. Jong:** Conceptualization; funding acquisition; methodology; validation; formal analysis; supervision; writing – review and editing. **Sarah Hanson:** Conceptualization; investigation; funding acquisition; methodology; writing – review and editing; supervision. **Shamima Akter:** Methodology; formal analysis; writing – review and editing. **Nikki Garner:** Formal analysis; writing – review and editing. **Wendy Hardeman:** Conceptualization; investigation; funding acquisition; methodology; validation; visualization; writing – review and editing; supervision.

## CONFLICT OF INTEREST STATEMENT

JM, STJ and WH declare funding by the University of East Anglia Faculty of Medicine and Health Sciences for the PhD studentship, of which this rapid review is a component. AMM declares grants from the following: AppleTree, The APPLE Tree programme: Active Prevention in People at risk of dementia through Lifestyle, bEhaviour change and Technology to build REsiliEnce, and NuBrain: UK Consortium for Optimal Nutrition for Healthy Brain Ageing. AMM declares support for attending meetings and/or travelling to deliver Nutrition Society presentations in London and Coventry, UK. WH declares she is a past President of the European Health Psychology Society (2022–2025), unpaid. SH, SA and NG have no conflicts of interest to declare. Study Registration Number in PROSPERO: CRD42024572262.

## Supporting information


File S1:

File S2:

File S3:

File S4:

## Data Availability

The data that support the findings of this study are available from the corresponding author upon reasonable request.
