# Supplementary material for: Understanding healthy eating and physical activity community‐centred behaviour change interventions for underserved populations: A mixed methods rapid review
Source: Br J Health Psychol. 2025 Dec 16;31(1):e70043. doi: 10.1111/bjhp.70043 (PMC12706567; doi:10.1111/bjhp.70043)
Supplement: Supplementary file 2 — File S2: [file BJHP-31-0-s004.docx]

**Supplementary File S2: Sample Search String**

Database: Ovid Medline

Date Searched: 22nd May 2024

Records Retrieved: 5276

1 eating.ab. 93811

2 diet*.ab. 646121

3 nutrition.ab. 168822

4 fruit.ab. 87660

5 vegetable*.ab. 71973

6 "plant based".ab. 10105

7 physical activit*.ab. 151488

8 exercis*.ab. 336540

9 sedentary.ab. 40296

10 sport*.ab. 94271

11 swimming.ab. 32962

12 danc*.ab. 7993

13 walk*.ab. 145931

14 run*.ab. 234749

15 1 or 2 or 3 or 4 or 5 or 6 or 7 or 8 or 9 or 10 or 11 or 12 or 13 or 14 1726636

16 communit*.ti. 222902

17 "third sector".ti. 53

18 "voluntary sector".ti. 85

19 "voluntary group".ti. 5

20 "voluntary organi*".ti. 95

21 "service group".ti. 26

22 "service program*".ti. 459

23 initiative.ti. 17067

24 "social prescrib*".ti. 271

25 16 or 17 or 18 or 19 or 20 or 21 or 22 or 23 or 24 240029

26 "under served".ab. 511

27 "under-served".ab. 511

28 "underserved".ab. 13734

29 "underrepresented".ab. 13056

30 minorit*.ab. 91016

31 BME.ab. 3368

32 BAME.ab. 319

33 ethnic*.ab. 182907

34 racial*.ab. 61838

35 poverty.ab. 32548

36 SES.ab. 22814

37 "low socioeconomic".ab. 10024

38 "socioeconomic".ab. 121650

39 "social class*".ab. 9546

40 "vulnerable adult".ab. 37

41 "vulnerable person".ab. 54

42 "vulnerable people".ab. 999

43 inequal*.ab. 42742

44 deprived.ab. 31426

45 disadvantaged.ab. 17962

46 low* income.ab. 54178

47 "low* social".ab. 7201

48 impoverish*.ab. 5556

49 "low* education".ab. 12055

50 disabilit*.ab. 240068

51 "learning difficult*".ab. 2728

52 "special need*".ab. 4452

53 "additional need*".ab. 539

54 refugee*.ab. 12492

55 asylum.ab. 3523

56 immigrant*.ab. 27473

57 migrant*.ab. 22133

58 "sex work*".ab. 7558

59 LGBT*.ab. 4115

60 lesbian*.ab. 8689

61 gay.ab. 13715

62 bisexual*.ab. 12324

63 transexual*.ab. 55

64 transgender*.ab. 11359

65 "sexual orientation".ab. 7570

66 veteran*.ab. 41751

67 homeless*.ab. 12754

68 traveller*.ab. 4563

69 rural.ab. 160108

70 "digital exlcu*".ab. 0

71 "digital divide".ab. 974

72 "mental* ill*".ab. 42357

73 "mental disorder".ab. 10591

74 "psychiatric illness".ab. 7040

75 "cognitive impair*".ab. 91059

76 "drug user*".ab. 15662

77 alcoholism.ab. 19904

78 26 or 27 or 28 or 29 or 30 or 31 or 32 or 33 or 34 or 35 or 36 or 37 or 38 or 39 or 40 or 41 or 42 or 43 or 44 or 45 or 46 or 47 or 48 or 49 or 50 or 51 or 52 or 53 or 54 or 55 or 56 or 57 or 58 or 59 or 60 or 61 or 62 or 63 or 64 or 65 or 66 or 67 or 68 or 69 or 70 or 71 or 72 or 73 or 74 or 75 or 76 or 77 1196520

79 review.ti. 758825

80 protocol.ti. 86763

81 15 and 25 and 78 5570

82 79 or 80 833550

83 81 not 82 5276
