# Supplementary material for: Understanding healthy eating and physical activity community‐centred behaviour change interventions for underserved populations: A mixed methods rapid review
Source: Br J Health Psychol. 2025 Dec 16;31(1):e70043. doi: 10.1111/bjhp.70043 (PMC12706567; doi:10.1111/bjhp.70043)
Supplement: Supplementary file 4 — File S4: [file BJHP-31-0-s003.docx]

Supplementary Material S4 - BCTO Frequency Mapping

**Intervention1: Active Mums - Arevalo (2023) (25 BCTs) [39]**

Level 1 (8 BCTs):

- Goal Directed
- Monitoring Behaviour
- Social support
- Guide how to perform behaviour
- Increase awareness of consequences
- Advise specific behaviour
- Advise how to change emotions
- Prompt focus on self identity

Level 2 (14 BCTs):

- Goal setting
- Goal strategizing
- Review behaviour
- Attend to discrepancy between current behaviour and goal
- Create behavioural contract
- Record behaviour without feedback
- Self monitor behaviour
- Provide feedback
- Advise to seek support
- Instruct how to perform behaviour
- Suggest how to perform behaviour
- Inform about health consequences
- Advise how to reduce negative emotions
- Identify self as a role model

Level 3 (3 BCTs):

- Set behaviour goal
- Advise to seek informational support
- Inform about positive health consequences

**Intervention 2. Community Garden – Chauvenet 2022 (2 BCTs) [40]**

Level 1 (1 BCT):

- Social support

Level 3 (1 BCT):

- Deliver instrumental support

**Intervention 3. Heart Matters – Frerichs 2020 (15 BCTs) [41]**

Level 1 (6 BCTs):

- Goal directed
- Monitoring behaviour
- Social support
- Guide how to perform behaviour
- Restructure the environment
- Prompt focus on self-identity

Level 2 (5 BCTs):

- Goal setting
- Self monitor outcome of behaviour
- Advise to seek support
- Arrange support
- Suggest how to perform behaviour

Level 3 (4 BCTs):

- Set behaviour goal
- Advise to seek instrumental support
- Advise to seek emotional support
- Arrange emotional support

**Intervention 4. Irish Mental Health Initiative – Gallagher 2020 (12 BCTs) [42]**

Level 1 (5 BCTs):

- Social support
- Guide how to perform behaviour
- Increase awareness of behaviour
- Awareness of other people’s thought, feelings and actions
- Advise specific behaviour

Level 2 (7 BCTs):

- Arrange support
- Deliver support
- Instruct how to perform behaviour
- Suggest how to perform behaviour
- Demonstrate the behaviour
- Increase salience of behaviour
- Suggest to change behaviour

**Intervention 5. Pedal Away – Hayton 2019 (5 BCTs) [43]**

Level 1 (2 BCTs):

- Guide how to perform behaviour
- Restructure the environment

Level 2 (3 BCTs):

- Instruct how to perform behaviour
- Demonstrate the behaviour
- Restructure the physical environment

**Intervention 6. With Every Step, We Grow Stronger – Lai 2019 (12 BCTs) [44]**

Level 1 (4 BCTs):

- Goal directed
- Monitoring behaviour
- Social support
- Guide how to perform behaviour

Level 2 (6 BCTs):

- Goal setting
- Record behaviour without feedback
- Self monitor behaviour
- Self monitor outcome of behaviour
- Advise to seek support
- Suggest how to perform behaviour

Level 3 (2 BCTs):

- Set behaviour goal
- Affirm commitment

**Intervention 7. N.Ireland PA initiative – Lawlor 2019 (29 BCTs) [45]**

Level 1 (7 BCTs):

- Goal directed
- Social support
- Guide how to perform behaviour
- Suggest different perspective on behaviour
- Increase awareness of consequences
- Prompt thinking related to successful performance
- Prompt focus on self-identity

Level 2 (13 BCTs):

- Goal setting
- Goal strategising
- Arrange support
- Deliver support
- Advise to seek support
- Suggest how to perform behaviour
- Instruct how to perform behaviour
- Inform about antecedents
- Inform about health consequences
- Inform about social consequences
- Inform about environmental consequences
- Inform about emotional consequences
- Consider pros and cons

Level 3 (9 BCTs):

- Arrange instrumental support
- Arrange emotional support
- Advise to seek instrumental support
- Advise to seek emotional support
- Inform about positive health consequences
- Inform about negative health consequences
- Inform about positive social consequences
- Inform about positive environmental consequences
- Inform about positive emotional consequences

**Intervention 8. MySteps – Luft 2023 (24 BCTs) [46]**

Level 1 (9 BCTs):

- Goal directed
- Monitoring behaviour
- Social support
- Guide how to perform behaviour
- Suggest different perspective on behaviour
- Increase awareness of behaviour
- Advise specific behaviour
- Manage mental processes
- Prompt thinking related to successful performance

Level 2 (12 BCTs):

- Goal setting
- Goal strategising
- Review behaviour goal
- Review behaviour goal plan
- Attend to discrepancy between current behaviour and goal
- Advise to keep behaviour goal in mind
- Provide feedback
- Deliver support
- Instruct how to perform behaviour
- Increase salience of behaviour
- Enable person to manage automatic responses
- Remind about personal capability

Level 3 (3 BCTs):

- Set behaviour goal
- Provide feedback on behaviour
- Deliver emotional support

**Intervention 9. The Circle of Friends Walking Programme - Martin Hammond 2022 (20 BCTs) [47]**

Level 1 (10 BCTs):

- Goal directed
- Monitoring behaviour
- Social support
- Guide how to perform behaviour
- Suggest different perspective on behaviour
- Increase awareness of behaviour
- Increase awareness of consequences
- Associative learning
- Prompt thinking related to successful performance
- Prompt focus on self-identity

Level 2 (6 BCTs):

- Goal setting
- Self-monitor behaviour
- Provide feedback
- Advise to seek support
- Suggest how to perform behaviour
- Increase salience of behaviour

Level 3 (3 BCTs):

- Set behaviour goal
- Provide feedback on behaviour
- Prompt Interventionended action

Level 4 (1 BCT):

- Set measurable behaviour goal

**Intervention. 10 African-Caribbean Eating Resources - Moore 2023 (5 BCTs) [48]**

Level 1 (2 BCTs):

- Guide how to perform behaviour
- Prompt thinking related to successful performance

Level 2 (3 BCTs):

- Instruct how to perform behaviour
- Suggest how to perform behaviour
- Demonstrate the behaviour

**Intervention 11. Parkrun Prove Project - Quirk & Haake 2019 & Quirk & Haake 2021 (28 BCTs) [49,50]**

Level 1 (8 BCTs):

- Monitoring behaviour
- Social support
- Suggest different perspective on behaviour
- Increase awareness of behaviour
- Increase awareness of consequences
- Awareness of other people’s thoughts, feelings and actions
- Restructure the environment
- Prompt focus on self identity

Level 2 (13 BCTs):

- Provide feedback
- Advise to seek support
- Deliver support
- Reattribute cause
- Increase salience of behaviour
- Inform about health consequences
- Increase salience of consequences
- Prompt comparative imagining of future outcomes
- Present information from credible influence
- Suggest to change behaviour
- Tell to change behaviour
- Implore to change behaviour
- Adopt positive self identity

Level 3 (7 BCTs):

- Advise to seek instrumental support
- Advise to seek emotional support
- Advise to seek informational support
- Deliver instrumental support
- Deliver emotional support
- Deliver informational support
- Inform about positive health consequences

**Intervention 12. Parkrun Outreach Ambassadors Project – Quirk 2024 (13 BCTs) [51]**

Level 1 (6 BCTs):

- Social support
- Guide how to perform behaviour
- Suggest different perspective on behaviour
- Increase awareness of behaviour
- Advise specific behaviour
- Restructure the environment

Level 2 (6 BCTs):

- Deliver support
- Instruct how to perform behaviour
- Inform about antecedents
- Increase awareness of option of novel behaviour
- Increase salience of behaviour
- Substitute behaviour

Level 3 (1 BCT):

- Deliver informational support

**Intervention 13. Eat Healthy, Be Active Community Workshops – Sanchez 2021 (2 BCTs) [52]**

Level 1 (1 BCT):

- Guide how to perform behaviour

Level 2 (1 BCT):

- Instruct how to perform behaviour

**Intervention 14. Pio Keeps Moving – Sanz-Remacha 2021, 2022, 2023 (47 BCTs) [53-55]**

Level 1 (14 BCTs):

- Goal directed behaviour
- Monitoring behaviour
- Social support
- Guide how to perform behaviour
- Increase awareness of behaviour
- Increase awareness of consequences
- Awareness of other people’s thoughts, feelings and actions
- Advise specific behaviour
- Manage mental processes
- Prompt thinking related to successful performance
- Advise to change emotions
- Restructure the environment
- Prompt focus on self identity
- Behavioural consequence

Level 2 (20 BCTs):

- Goal setting
- Provide feedback
- Advise to seek support
- Arrange support
- Deliver support
- Instruct how to perform behaviour
- Suggest how to perform behaviour
- Demonstrate the behaviour
- Re-attribute cause
- Increase awareness of option of novel behaviour
- Increase salience of behaviour
- Inform about health consequences
- Consider pros and cons
- Advise behavioural ways to change emotions
- Restructure the social environment
- Restructure the physical environment
- Increase awareness of other’s approval
- Practice behaviour
- Provide consequences for behaviour
- Present information from credible influence

Level 3 (13 BCTs):

- Set behaviour goal
- Provide feedback on behaviour
- Advise to seek instrumental support
- Advise to seek emotional support
- Advise to seek informational support
- Arrange instrumental support
- Arrange emotional support
- Arrange informational support
- Arrange appraisal support
- Deliver instrumental support
- Deliver emotional support
- Deliver informational support
- Provide positive consequence for behaviour

**Intervention 15. Stronger Austin - Springer 2022 (11 BCTs) [56]**

Level 1 (4 BCTs):

- Social support
- Guide how to perform behaviour
- Restructure the environment
- Awareness of other people’s thoughts, feelings and actions

Level 2 (5 BCTs):

- Deliver support
- Instruct how to perform behaviour
- Suggest how to perform behaviour
- Restructure the physical environment
- Present information from credible source

Level 3 (2 BCTs):

- Deliver emotional support
- Deliver informational support

**Intervention 16. Malmo Migrant Interventionervention - Ramji 2022 (14 BCTs) [57]**

Level 1 (6 BCTs):

- Social support
- Increase awareness of behaviour
- Prompt thinking related to successful performance
- Prompt focus on self identity
- Outcome consequence
- Awareness of other people’s thoughts, feelings and actions

Level 2 (5 BCTs):

- Advise to seek support
- Arrange support
- Affirm valued self-identity
- Provide consequence for outcome of behaviour
- Present information from credible source

Level 3 (3 BCTs):

- Advise to seek emotional support
- Arrange emotional support
- Provide positive consequence for outcome of behaviour

**Intervention 17. Women Warrior Programme - Wicklum 2019 (24 BCTs) [58]**

Level 1 (5 BCTs):

- Goal directed
- Monitoring behaviour
- Social support
- Guide how to perform behaviour
- Awareness of other people’s thoughts, feelings and actions

Level 2 (8 BCTs):

- Goal setting
- Advise to seek support
- Arrange support
- Deliver support
- Instruct how to perform behaviour
- Suggest how to perform behaviour
- Demonstrate the behaviour
- Present information from credible source

Level 3 (10 BCTs):

- Set behaviour goal
- Advise to seek instrumental support
- Advise to seek emotional support
- Advise to seek informational support
- Arrange instrumental support
- Arrange emotional support
- Arrange informational support
- Deliver instrumental support
- Deliver emotional support
- Deliver informational support

Level 4 (1 BCT):

- Set measurable behaviour goal

**Intervention 18. Indigenous Women PA programme – Wicklum 2023 (20 BCTs) [59]**

Level 1(4 BCTs):

- Goal directed
- Monitoring behaviour
- Social support
- Prompt focus on self identity

Level 2: (5 BCTs)

- Goal setting
- Advise to seek support
- Arrange support
- Deliver support
- Identify self as role mode

Level 3 (10 BCTs):

- Set behaviour goal
- Advise to seek instrumental support
- Advise to seek emotional support
- Advise to seek informational support
- Arrange instrumental support
- Arrange emotional support
- Arrange informational support
- Deliver instrumental support
- Deliver emotional support
- Deliver informational support

Level 4 (1 BCT):

- Set measurable behaviour goal
